# Clinical outcomes of elderly patients (≥70 years) with resectable esophageal squamous cell carcinoma who underwent esophagectomy or chemoradiotherapy: A retrospective analysis from a single cancer institute

**DOI:** 10.1097/MD.0000000000005630

**Published:** 2016-12-16

**Authors:** Wang Jing, Hongbo Guo, Li Kong, Yan Zhang, Haiyong Wang, Changchun An, Hui Zhu, Jinming Yu

**Affiliations:** a Department of Radiation Oncology, The First Affiliated Hospital of Zhengzhou University, Zhengzhou; b Department of Radiation Oncology; c Department of Thoracic Surgery, Shandong Cancer Hospital Affiliated to Shandong University; d Shandong Academy of Medical Sciences; e Department of Medical Oncology, Shandong Cancer Hospital Affiliated to Shandong University, Jinan, Shandong, China.

**Keywords:** chemoradiotherapy, elderly, esophageal cancer, prognosis factor, surgery, survival

## Abstract

A retrospective analysis was conducted to investigate outcomes of elderly patients with resectable esophageal squamous cell carcinoma (ESCC) who underwent surgery or chemoradiotherapy (CRT).

We performed a retrospective review of the records of elderly patients (≥70 years) with resectable ESCC who underwent esophagectomy or CRT between January 2009 and March 2013. According to the main treatment strategy, patients were allocated into either surgery group or CRT group. Overall survival (OS), cancer-specific survival and progression-free survival were calculated by the Kaplan–Meier method. Univariate and multivariate survival analyses were performed by the Kaplan–Meier method and Cox proportional hazards model, respectively.

A total of 188 patients were enrolled. Eighty-eight patients underwent esophagectomy, and 100 patients underwent CRT. The median age of the patients was 73 years (range, 70–81 years) in the surgery group and 76 years (range, 70–88 years) in the CRT group. The median survival time (MST) for the whole cohort was 25.6 months, and 1-, 3-, and 5-year survival rates were 69.2%, 36.1%, and 21.9%, respectively. The MST in the surgery group and the CRT group was 36 months and 15 months, respectively. The 1-, 3-, and 5-year survival rates in the surgery group were 82.4%, 49.0%, and 33.3%, compared to 58.0%, 24.1%, and 7.8% in the CRT group (*P* < 0.0001). Multivariate analysis revealed that lymph node status (hazard ratio [HR] = 0.598, *P* = 0.011) and treatment strategies (HR = 0.538, *P* = 0.001) were independent and significant prognostic factors for OS in elderly patients.

Surgery was the main treatment strategy for elderly patients with ESCC. Advanced age and comorbidities should not be the cause for elderly patients to avoid aggressive regimens. Delivered therapeutic approaches should be individualized on the basis of carefully evaluating the balance of benefits, risks, and life expectancy.

## 1 Introduction

Cancer has become the leading cause of death in the elderly aged from 60 to 79 years in the United States; approximately 70% of all cancers will be diagnosed in the elderly aged ≥65 years by 2030.^[1,2]^ Esophageal cancer (EC), which the incidence projected increased from 35% in 2020 to 68% in 2030, has become the fifth in male and eighth in female cause of cancer death worldwide; approximately 482,300 new cases and 406,800 deaths from EC occurred in 2008 worldwide.^[2,3]^ As actuarial life expectancy increases, it is extremely urgent to pay more attention to choose optimal anticancer strategy in the elderly. In addition, with the establishment of evidence-based medicine, especially the advent of precision medicine, the decision of treatment options is based on evidence and paid more attention on individual. Unfortunately, the elderly was often underrepresented in clinical trials considering advanced age and comorbidities. As a result, high levels of evidences are shortage to guide the choice of treatment for elderly cancer patients. To date, there is no standardized treatment strategy for elderly patients with EC.

Surgery remains the main approach to treat patients with EC. However, high rates of postoperative morbidity and mortality occurred in patients aged ≥70 years who treated with esophagectomy.^[4]^ Furthermore, considering high risk factors of surgery, such as advanced age and comorbidities, the approach of surgery was not being chosen or refused by patients themselves and their families. Hence, for patients with high risk of postoperative morbidity, concurrent chemoradiotherapy (CRT) was the standard treatment regimen. Regardless of similar response rate and overall survival (OS) compared with younger populations treated with CRT, the survival rate remains poor with approximately 25% to 64% of 2-year OS rate in the elderly. Thus, for elderly patients with potentially resectable thoracic EC, comparison of outcomes between surgery and CRT is very necessary.

The modality of treatment that elderly patients could benefit from remains unknown. Hence, we conducted a retrospective study to analyze the prognostic factors of elderly patients with nonmetastatic EC, who treated with different treatment strategies (surgery, CRT, and combination therapies), and attempted to afford new evidences of choosing optimal approaches in such patients.

## 2 Patients and methods

### 2.1 Patients

We retrospectively reviewed the records of patients with EC who underwent surgery or CRT in our cancer department between January 2009 and March 2013. The criteria of enrollment were treatment-naïve patients with histologically proven thoracic esophageal squamous cell carcinoma (ESCC); aged ≥70 years; KPS ≥70; clinical stage T1-3; complete resection (R0); without any other site of carcinoma or with metastatic disease. Complete records showed that patients were suitable for surgery without serious cardiopulmonary comorbidities after cautious assessment. Comorbidities were evaluated by the Charlson criteria system.^[5]^ Clinical tumor stage and status of lymph node (LN) were evaluated by computed tomography (CT) scan, according to the criteria of Union for International Cancer Control Tumor-Node-Metastasis cancer staging, 7th edition (2010). Tumor length was measured by esophagography before treatment. All data were analyzed anonymously. This study was approved by the institutional ethics committee of Shandong Cancer Hospital Affiliated to Shandong University.

### 2.2 Procedures

In our department, standard surgical procedures consisted of 2 types: for upper and middle sites, esophagectomy with mediastinal lymphadenectomy via left thoracotomy, upper abdominal lymphadenectomy, reconstruction of the gastric tube, and anastomosis above the aortic arch or in the cervical incision were recommended; for lower sites, laparotomy with mediastinal and abdominal lymphadenectomy, and anastomosis under the aortic arch were recommended. Whether cervical lymphadenectomy or not was based on the location of the primary tumor and invasion of regional LNs.

Radiotherapy was administered by 3-dimensional conformal radiotherapy or intensity modulated radiation therapy. For involved-field irradiation (IFI), the gross tumor volume (GTV) was defined any visible esophageal lesions measured by any available imaging examinations, including CT, barium esophagogram, positron emission tomography (PET)/CT and localizable CT, and any involved regional LNs. Clinical targeted volume (CTV) was defined as the GTV plus at least 3-cm margin superior and inferior to the primary tumor and a lateral margin 0.8 to 1.0 cm (for LN at least 0.5 cm). For elective nodal irradiation, GTV and CTV of primary tumor was defined similar with that of IFI, CTV of LN include involved LNs and related lymphatic drainage areas. Elective treatment of nodal regions depended upon the location of the primary tumor. Planning targeted volume was defined as the CTV plus a 0.5 to 1.0 cm radial margin. A single daily fraction of 1.8 to 2.0 Gy was administered, for a total dose of 50.4 to 66 Gy to the primary lesions and involved LNs, and 40 to 50.4 Gy for elective node irradiation.

Chemotherapy consisted of adjuvant chemotherapy and concurrent chemotherapy. Cisplatin (25 mg/m^2^, on days 1–3) and 5-fluorouracil (1000 mg/m^2^, on days 1–5)/docetaxel (75 mg/m^2^, on days 1) were administered every 3 weeks up to 2 to 4 cycles.

### 2.3 Mortality and complications

Short-term mortality is defined as death occurred during hospital admission or within 30 days of surgery. Complications consists of any grade postoperative complications, which was assessed using the criteria of modified Clavien-Dindo classification,^[6]^ and grade 3 or greater CRT adverse events, which was accessed using the CTCAE version 3.0. Infection included pyothorax or chylothorax, abdominal abscess, or incision infection. Pulmonary complications were defined as pneumonia, or hypoxia requiring intervention. Cardiac complications were defined as heart failure, myocardial infarction, or arrhythmia requiring intervention.

### 2.4 Follow-up

Cervical ultrasonography, chest and abdominal CT, and/or esophagography were performed to monitor the course of disease. For patients with surgery, CT scan was performed every 3 months up to 2 years, every 6 months up to 5 years, and annually thereafter. For patients with CRT, CT scan was performed every 3 months for the first year, and then every 6 months for the next 3 years, and annually thereafter. Other necessary follow-up evaluation included history, physical examination, complete blood count, and serum biochemistry. Another unscheduled evaluation was conducted according to the clinical situation.

### 2.5 Statistical analysis

Chi-square test was performed to compare the difference of patients’ characteristics. OS was measured from the date of diagnosis to the date of death from any cause or the last known follow-up date. Cause-specific survival (CSS) was defined as the interval from the date of treatment to death from any cause or the last follow-up. Progression-free survival (PFS) was calculated from the date of diagnosis to the date of disease progression or death from any cause, or to the date of censor. OS, CSS, and PFS were calculated using the Kaplan–Meier method, and the log-rank test was used to evaluate the difference in survival curves between different arms. Univariate survival analysis was performed by the Kaplan–Meier method. Multivariate analysis for OS was performed using Cox regression and a backward–forward stepwise method was selected. All variables with *P* values <0.10 in the univariate analysis were included in the multivariate analysis. All statistical tests were 2-tailed, and *P* < 0.05 was considered statistically significant.

## 3 Results

### 3.1 Patients characteristics

A total of 214 patients with ESCC who underwent esophagectomy or CRT in our cancer center were collected between January 2009 and March 2013. The median follow-up period for the whole cohort was 28.5 months (range, 0–81 months). At the end of the follow-up, 26 (12.1%) patients were lost, and 58 patients remained alive. Finally, 188 patients were enrolled in this study, including 88 patients in the surgery group and 100 patients in the CRT group, while the median age were 73.0 years (range, 70–81 years) and 76.0 years (range, 70–88 years), respectively. Of 88 patients treated with surgery, 63 patients underwent surgery alone, and the remaining 25 patients, including 10 patients of surgery in combination with chemotherapy, 11 patients of surgery in combination with radiotherapy, and 4 patients of surgery in combination with CRT. All characteristics of patients are listed in Table 1. Although the baseline data on LNs status showed a significant difference between the CRT group and the surgery group, the clinical stage before treatment were similar (data not show).

**Table 1:**
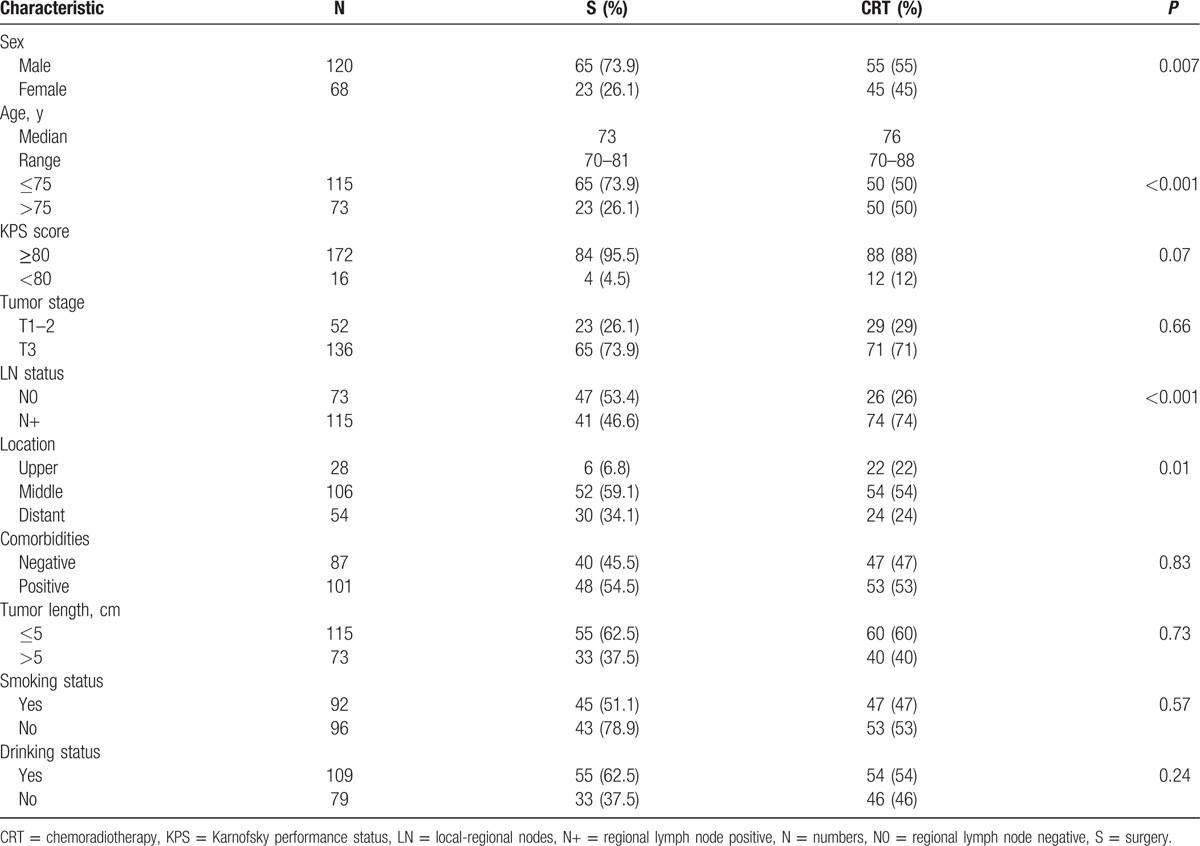
Clinical features of ESCC patients treated with surgery or CRT.

### 3.2 Survival

**Figure 1:**
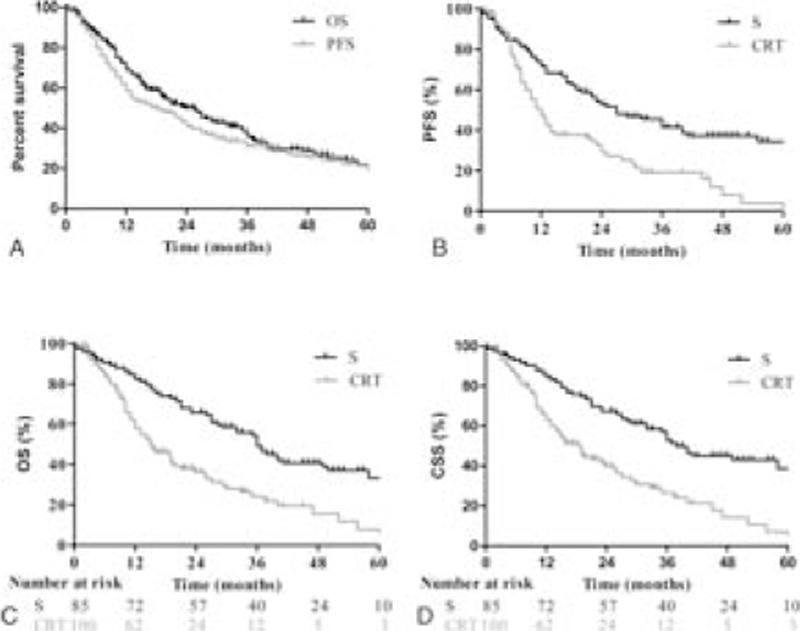
Survival curve for 188 elderly patients with esophageal cancer treated with surgery or chemoradiotherapy. (A) For entire group, the median progression-free survival (PFS) and median survival time (MST) were 18 months and 25.6 months, and 5-year survival rate was 21.9%; (B) the median PFS for surgery group and CRT group were 27 months and 12 months, respectively. 1-, 3-year PFS rates were 71.8% and 41.5% in S group, compared to 47.3% and 19.1% in the CRT group (*P* < 0.001); (C) The MST were 36 months and 15 months in the surgery group and CRT group; 1-, 5-year survival rates were 82.4% and 33.3%, and 58.0% and 7.8%, favoring surgery group (*P* < 0.001); (D) the median cancer-specific survival in the surgery group and CRT group were 38 months and 18.6 months, respectively; 5-year survival rate was 84.4% in the surgery group, compared to 26.5% in the CRT group (*P* < 0.001).

Figure 1A shows OS and PFS curve for entire group who underwent surgery and CRT. For the whole patients’ cohort, the median PFS was 18 months; 3- and 5-year PFS rates were 31.0% and 19.5%, respectively. The median survival time (MST) was 25.6 months, and 1-, 3-, and 5-year survival rates were 69.2%, 36.1%, and 21.9%, respectively.

Figure 1B shows PFS of patients between the surgery group and the CRT group. The median PFS for surgery group and CRT group were 27 months and 12 months, respectively. And, 1-, 2-, and 3-year PFS rates were 71.8%, 52.8%, and 41.5% in the surgery group, which were significant higher than that of 47.3%, 29.2%, and 19.1% in the CRT group (*P* < 0.0001).

Figure 1C shows the survival of patients who underwent surgery or CRT. For the surgery group, 1-, 3-, and 5-year survival rates were 82.4%, 49.0%, and 33.3%, whereas 58.0%, 24.1%, and 7.8% in the CRT group. The difference was significant with a *P* value of less than 0.0001. The MST was 36 months and 15 months for the surgery group and CRT group, respectively.

Figure 1D shows CSS of patients who underwent surgery or CRT. For the surgery group and CRT group, 1-, 3-, and 5-year survival rates were 84.4%, 67.5%, and 52.7%, and 63.0%, 39.9%, and 26.5%, respectively. The difference was significant with less than 0.0001 of *P* value. The MST in the surgery group was 38 months, whereas 18.6 months in the CRT group.

### 3.3 Prognostic factors

The patient characteristics evaluated to determine their prognostic value for OS are summarized in Table 2. Univariate analysis revealed that sex, age, tumor stage, location, tumor length, smoking status, and drinking status were not associated with survival; however, LN status (*P* = 0.000), comorbidities (*P* = 0.03), and treatment strategies (*P* = 0.000) were significant prognostic factors for survival. Multivariate analysis revealed that LN status (hazard ratio [HR] = 0.598, *P* = 0.011) and treatment strategies (HR = 0.538, *P* = 0.001) were independent and significant prognostic factors for OS in elderly patients.

**Table 2:**
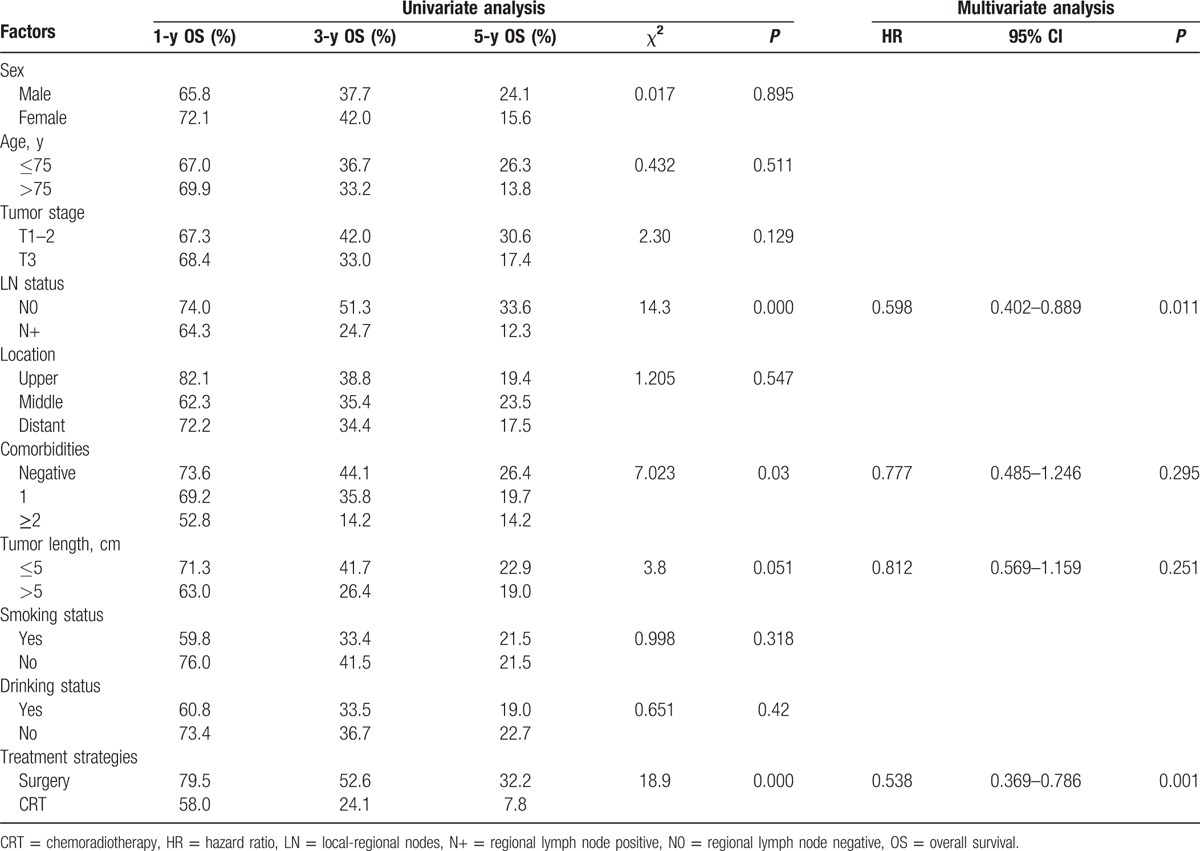
Univariate and multivariate analyses of the effect of prognostic factors on OS in patients with EC.

### 3.4 Morbidity and mortality

Treatment-induced toxicities are detailed in Table 3. As detailed in the surgery group, infection (12.5%) and anastomotic leakage (6.8%) were the main complications, whereas leucopenia (21%), esophagitis (12%), and pneumonia (10%) were in the CRT group.

**Table 3:**
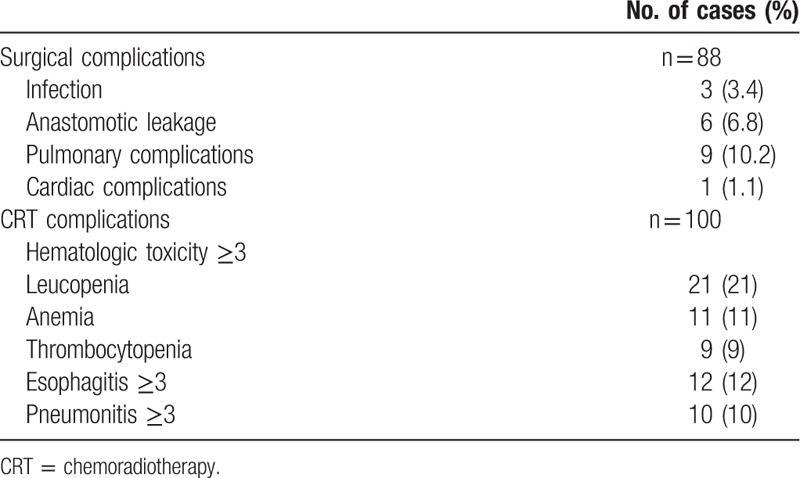
Side effects of patients who underwent surgery or CRT.

Table 4 shows mortality of patients treated with surgery and CRT. A total of 130 patients (54 in the surgery group and 76 in the CRT group) died of primary tumor or other causes. Of 54 deaths in the surgery group, 5 (9.3%) patients died of perioperative complications; 29 (53.7%) and 6 (11.1%) patients died of primary tumor and hemorrhage in long-term follow-up analysis, respectively. Only 4 patients died of nontumor-related cause, including 2 of secondary primary tumor, each case of cardiovascular complication and accident. For the CRT group, primary tumor (61.8%), radiation pneumonia (13.2%), and hemorrhage (9.2%) were the main causes of death. Of 7 patients with nontumor-related death, 4 patients died of pulmonary complications (n = 2) and cardiac complications (n = 2), and each case died of accident, cerebral hemorrhage, and miscellaneous complication.

**Table 4:**
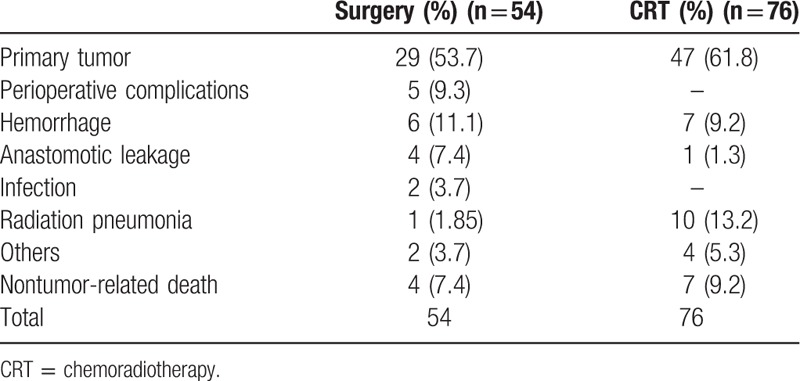
Mortality of patients who underwent surgery or CRT.

## 4 Discussion

EC is one of the most common causes of cancer deaths worldwide, and the incidence has been rising in recent years.^[3]^ Squamous cell carcinoma is the most common histology of EC in Asia. With quickening the population aging, newly diagnosed EC in elderly patients increased dramatically. However, the optimal strategy for the elderly was still inconsistent. Hence, we conducted a retrospective study to compare survival and adverse events in elderly patients with ESCC who underwent surgery or CRT. Clinical features but not LN status and tumor location in the surgery and CRT group were well balanced. More positive LNs distributed in the CRT group, and multivariate analysis demonstrated positive LNs was a negative prognostic factor on survival. For patients with surgery, 5-year survival rate was significant higher than that of patients with CRT (33.3% vs 7.8%. *P* = 0.0001). Furthermore, perioperative and postoperative complications and mortality, and the CRT-induced toxicities were also acceptable. Advanced age and comorbidities have no influence on long-term survival.

Age, probably is the main cause to affect the treatment strategy administered to elderly patients. Generally, patients aged ≥75 years were always excluded by randomized trials. The prognostic value of advanced age was still controversial.^[7–9]^ A retrospective study including 722 patients with thoracic EC, who underwent esophagectomy with or without neoadjuvant therapy, indicated that the 5-year OS rate of patients in aged <70-years group was similar with that in aged 70 to 75-years group (52.4% vs 50.2%), but significant higher than that in aged 75 to 80-year group (52.4% vs 38.1%, *P* = 0.011).^[7]^ However, the 5-year CSS in aged <70, 70 to 75, and 75 to 80-year groups was no significant difference (57.9%, 61.7%, and 52.4%, respectively). For patients with aged >80 years, the survival was significant lower than patients aged ≤80 years. Another retrospective analysis also indicated worse outcomes in octogenarians but showed a better 5-year survival in patients aged <70 years than that in patients aged 70 to 79 years (64.8% vs 41.7%, *P* = 0.006).^[8]^ Regardless of low mortality concluded in both of 2 studies, whether age is a prognostic factor or not is still inconsistent. A tendency that worse survival in patients aged ≥80 years rather than patients aged <80 was observed. In present study, advanced age was not demonstrated as a prognostic factor on survival (*P* = 0.511). However, no patients aged ≥80 years were concluded in present study.

Surgery remained the best modality to treat solid tumors, even though advanced age was an important consideration but not the sole factor that may affect the prognosis.^[9]^ Outcomes of elderly patients with surgery were promised.^[7,8,10–13]^ A meta-analysis reviewed 25 publications including 2573 elderly patients (aged ≥70 years) treated with esophagectomy; the results showed that 5-year OS and CSS were 21.23% and 34.4%.^[11]^ Relatively low survival may attribute to microscopic residual/macroscopic residual disease resection and clinical stage IV in patients’ cohort. In present study, all patients underwent a complete resection, and 5-year OS rate was 33.3%. The data from Shimada's study indicated that 5-year OS rate reached 57% in patients aged ≥75 years who underwent esophagectomy.^[12]^ Emerging data from clinical studies indicated that the 5-year CSS rate was approximately 41.5% to 61.7% in elderly patients aged ≥70 years treated with surgery.^[7]^ The survival of patients with CRT instead of surgery was low.^[14]^ A data from China indicated that the 3-year OS rate was only 23.2% with a short MST of 17.9 months in elderly patients with ESCC aged ≥70 years who underwent definitive radiotherapy or CRT.^[15]^ Even for patients with potentially resectable disease, 5-year survival rate was only 17% with a low rate of R0 resection (53%).^[16]^ Similarly, our data also demonstrated that 7.8% of 5-year survival was lower in elderly patients treated with CRT than that with surgery. Therefore, highly evaluated candidates who can benefit from operation should be given surgery firstly.

Previous studies indicated that postoperative complications and the presence of increased comorbidities reduced the survival of patients. The data from Cijs's study that included 250 patients aged ≥70 years who underwent esophagectomy showed high surgical complications (20%), nonsurgical complications (35%), and operative mortality (8.4%), which may decrease 5-year disease-specific survival to 27%.^[10]^ Another study also indicated that the incidence of postoperative complications in patients aged 70 to 79 years was significant higher than that of young patients aged ≤69 years (42% vs 32%, *P* < 0.05), and 5-year survival rate was worse in patients aged 70 to 79 years than that patients aged <70 years (29% vs 38%, *P* < 0.01).^[13]^ Adverse events for patients with surgery were different from that of patients with CRT. Pulmonary complications and anastomotic leakage were the main treatment-related toxicities for surgery, whereas hematologic toxicities, radiation pneumonia, and radiation esophagitis were for CRT. Despite our previous study has demonstrated that the CRT-induced toxicities were well tolerated, the rate of radiation-induced pneumonia was still high (10.2%) for elderly patients.^[17]^ However, postoperative complications in present study, mainly pulmonary complications (10.2%) and anastomotic leakage (6.8%), were lower than that of previous studies. The decreased postoperative complications may be attributed to the improvement of surgical technique and postoperative nursing management. Moreover, the CRT-induced toxicities were also acceptable. Comorbidities were likely to increase the risk of treatment toxicities and even treatment-induced death for patients.^[18]^ A large-scaled observation study including 17,712 patients showed worse survival in patients with more sever levels of comorbidity.^[19]^ Another large-scaled retrospective study demonstrated that comorbidity was an independent prognostic factor for patients with cancer.^[19]^ However, multivariate analysis in present study revealed that comorbidities were not the prognostic factor on survival. However, more comorbidities were likely to contribute to the lower performance status that might increase the risk of postoperative complications. Considering inconsistent evidences of complications and comorbidities on survival, comorbidities and the risk of postoperative complications should be evaluated carefully to perform the individualized treatment.

A notable issue in present study is that the status of LNs was not well balanced between the 2 groups. Previous studies indicated the nodal status, particularly the number of involved LNs, influenced the survival of patients with EC.^[20–22]^ Patients with negative LNs have the best prognosis, whereas patients with more than 4 involved LNs had the similar poor survival with that of patients with metastatic disease.^[20]^ In Wang's study, the survival of patients with regional lymph node negative (pN0) was significantly higher than that of patients with pN1-3; and the more involved LNs, the worse prognosis (pN1 vs pN2, *P* = 0.001; pN2 vs pN3, *P* < 0.001).^[21]^ The survival of patients with N0 was superior to those patients with positive LNs with 46% of 4-year survival rate in N0 disease, while 21% in N1 disease and 0% in N2 disease (*P* < 0.01).^[22]^ Our results were consistent with previous studies. However, more regional lymph node positive patients were in the CRT group than in the surgery group (74.0% vs 46.6%, *P* < 0.001), which may result in a worse survival rate for patients in the CRT group than in the surgery group. Pathological LN status can only be available in the surgery group, whereas in the CRT group, LN status can only be established by CT and other imaging modalities. In China, LNs enlargement can be otherwise induced from air pollution, chronic bronchitis, and tuberculosis, these LNs can be diagnosed as “positive” on CT or PET imaging. In our clinical practice, the treatment strategy (surgery or CRT) is often based on the CT or PET imaging. In this study, clinical LN status between the 2 groups was well paired (not listed). Surgery can provide accurate disease staging to guide postoperative treatment, which would be advantageous for the better survival in the surgery group. Other limitations in this retrospective study were as follows: First, the inherent selection bias of retrospective analysis was not ruled out. Second, the samples were too small to analyze more potential impacts of OS, and on the other hand, too small samples result in the imbalanced data to a certain extent between the 2 groups on clinical characters. Expanding the scope of the time or the sample size may reduce the bias. Third, endoscopic ultrasonography was not as a routine examination in our cancer center, as a result, accurate Tumor/Node stage cannot be offered. According to the final results, the shortages do not affect the final research conclusion.

## 5 Conclusion

Although the heterogeneity of LN status weakens the reliability of study, it can still conclude that surgery was the main treatment strategy for elderly patients with potentially resectable ESCC, with an acceptable rate of perioperative mortality. For surgical patients, the risks and benefits should be carefully assessed based on life expectancy. Owing to the accurate postoperative stage, patients in the surgery group can benefit from the adjuvant therapeutic modalities after surgery. For this reason, highly selected elderly patients with resectable disease should be actively treated with surgery. Advanced age and comorbidities were not independently impact the decision of surgery. In consideration of the complex situation of elderly patients, individual treatment is urged. For patients who medically inoperable or decline surgery, definitive CRT remains the main approach to prolong survival. However, as a retrospective study, it may limit the generalizability of our conclusion. Further investigations with large-scale, prospective are warranted.
